# Lactoferrin Affects the Viability of Bacteria in a Biofilm and the Formation of a New Biofilm Cycle of *Mannheimia haemolytica* A2

**DOI:** 10.3390/ijms25168718

**Published:** 2024-08-09

**Authors:** Lucero Ruiz-Mazón, Gerardo Ramírez-Rico, Mireya de la Garza

**Affiliations:** 1 Departamento de Biología Celular, Centro de Investigación y de Estudios Avanzados del Instituto Politécnico Nacional (CINVESTAV-IPN), Ciudad de Mexico 07360, Mexico; lucero.ruiz@cinvestav.mx (L.R.-M.); garmvz@gmail.com (G.R.-R.); 2 Facultad de Estudios Superiores Cuautitlán, Universidad Nacional Autónoma de México (UNAM), Km 2.5 Carretera Cuautitlán-Teoloyucan, Cuautitlán Izcalli 54714, Mexico

**Keywords:** *Mannheimia haemolytica*, biofilm, bovine Lactoferrin, confocal laser microscopy

## Abstract

Respiratory diseases in ruminants are responsible for enormous economic losses for the dairy and meat industry. The main causative bacterial agent of pneumonia in ovine is *Mannheimia haemolytica* A2. Due to the impact of this disease, the effect of the antimicrobial protein, bovine lactoferrin (bLf), against virulence factors of this bacterium has been studied. However, its effect on biofilm formation has not been reported. In this work, we evaluated the effect on different stages of the biofilm. Our results reveal a decrease in biofilm formation when bacteria were pre-incubated with bLf. However, when bLf was added at the start of biofilm formation and on mature biofilm, an increase was observed, which was visualized by greater bacterial aggregation and secretion of biofilm matrix components. Additionally, through SDS-PAGE, a remarkable band of ~80 kDa was observed when bLf was added to biofilms. Therefore, the presence of bLf on the biofilm was determined through the Western blot and Microscopy techniques. Finally, by using Live/Dead staining, we observed that most of the bacteria in a biofilm with bLf were not viable. In addition, bLf affects the formation of a new biofilm cycle. In conclusion, bLf binds to the biofilm of *M. haemolytica* A2 and affects the viability of bacteria and the formation a new biofilm cycle.

## 1. Introduction

*M. haemolytica* is an inhabitant of the nasopharynx and tonsils of cattle and ovine, but under some risk factors such as viral infections or the transport of animals between farms, it can induce acute fibronecrotizing bronchopneumonia and pleuritis; this disease is known as mannheimiosis [1,2]. Respiratory diseases in ruminants are responsible for enormous economic losses for the dairy and meat industries, of approximately USD 1 billion per year in the USA [3]. The main causative bacterial agent of pneumonia in ovine is *M. haemolytica* serotype A2 [4]. Resistance genes to multiple antimicrobial groups have been found in *M. haemolytica* A2, such as to sulphonamides, tetracyclines, phenicols, and aminoglycosides [5]. *M. haemolytica* possesses some factors and mechanisms of pathogenicity to survive, colonize, and cause disease [6,7,8]. Among these, it can form biofilms, an important mechanism of pathogenicity that protects and helps bacterial cells to subsist inside the host. Biofilms are a complex community of aggregated bacteria adhered or unattached to a surface [9]. This structure allows bacteria to survive harsh environmental conditions, such as starvation, desiccation, and changes in temperature and pressure, and also protects them from host immune system response and bacteriophages. Biofilms also protect bacteria against some antibiotics, leading to antibiotic resistance and bacterial infections [10,11].

The biofilm of *M. haemolytica* serotype A1 has been characterized by Boukahil et al., who determined its main macromolecular constituents. In this study, the authors showed that proteins are the major component of the biofilm at a concentration of 9.7 μg/cm^2^, followed by carbohydrates and DNA, with concentrations of 0.87 μg/cm^2^ and 0.47 μg/cm^2^, respectively [12].

Due to the impact of biofilms on bacterial infections, some antibiofilm strategies have been evaluated, such as the use of molecules targeting components of the biofilm matrix, antiadhesion agents, quorum sensing inhibitors, nanoparticles, and others [13,14,15,16]. However, biofilms have developed resistance and tolerance mechanisms to conventional antimicrobials, making developing antibiofilm strategies a hard challenge [17]. On the other hand, biosafety, efficacy, and treatment costs are excessive. Due to this and antimicrobial resistance to antibiotics, a promising alternative could be lactoferrin.

Lactoferrin, a glycoprotein of the innate immune system, has been studied for its antimicrobial effect [18]. In particular, the effect of bLf on the inhibition of biofilm formation has been reported in many bacteria [19,20,21,22,23]; however, there are some reports that show a stimulatory effect on bacterial biofilms, such as *Streptococcus mutans* [24], *A. pleuropneumoniae* [25], and *Escherichia coli* [26]. The authors reported that the increase in biofilms by Lf could be due to a stress response, as this effect is observed with sub-lethal concentrations of Lf, which probably cause bacteria to stimulate a protective mechanism such as biofilm formation. Our group has reported the effect of bovine lactoferrin (bLf) on leukotoxins, lipopolysaccharides, outer membrane vesicles (OMVs), and the secretion of proteases from *M. haemolytica* serotype A2 [27,28]; however, the effect on the biofilm formation of this bacterium has not been analyzed yet. The aim of this work was to evaluate the effect of bLf on the biofilm formation of *M. haemolytica* A2. The results show a decrease in biofilm formation when bacteria were pre-incubated with sub-inhibitory concentrations of bLf; however, when bLf was added at the beginning of biofilm formation or to a mature biofilm, biofilm formation increased. However, the presence of bLf on the biofilm was confirmed, and dead bacteria from a biofilm with bLf were observed to a greater extent than bacteria from a biofilm with no treatment. Additionally, the formation of a new biofilm cycle was affected, since bacteria from a biofilm with bLf formed less biofilm than bacteria derived from a biofilm without bLf.

## 2. Results

### 2.1. Effect of Sub-Inhibitory Concentrations of bLf on the Growth of M. haemolytica A2

**Figure 1:**
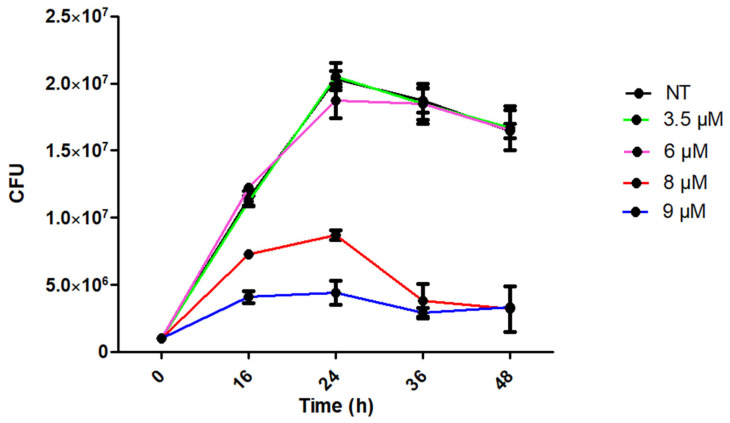
**Viability of *M. haemolytica* A2 with bLf.** *M. haemolytica* incubated with different concentrations of bLf at different times. The concentrations of 3.5 and 6 μM were sub-inhibitory until the 48th hour of incubation. Concentrations of 8 and 9 μM were inhibitory since they showed a significant difference regarding bacteria grown without bLf (NT) *p* < 0.05. Representative results of three independent experiments.

To determine the effect of bLf on the biofilm formation of *M. haemolytica* A2, it is necessary to use concentrations of bLf that will not kill the bacteria during the time required for biofilm formation. To this end, we first tested the effect of bLf on the growth of *M. haemolytica* A2 (10^6^ CFU) grown with different concentrations of bLf (0, 3.5, 6, 8, and 9 μM), at 16, 24, 36, and 48 h. The graph shows the viability of bacteria with or without bLf; at concentrations of 3.5 and 6 μM, there was no difference in bacterial growth regarding bacteria without bLf in all tests, while at concentrations of 8 and 9 μM, there was a significant decrease in bacterial growth from the first few hours (Figure 1). With this result, we concluded that the sub-inhibitory concentrations of bLf to *M. haemolytica* A2 at the times analyzed here are 3.5 and 6 μM. Then, to observe the effect of bLf on the biofilm formation of this bacterium, these concentrations were used in bacterial cultures of 10^6^ CFU for the following experiments.

### 2.2. Biofilm Formation of M. haemolytica A2 Is Negatively Affected When Bacteria Are Pre-Incubated with bLf but Increases When bLf Is Added during the Formation of Biofilm

**Figure 2:**
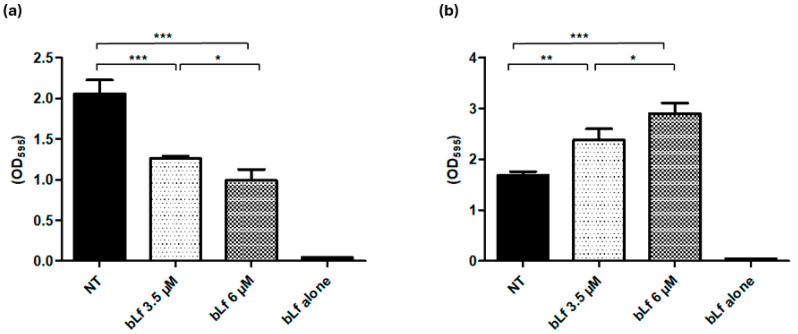
**Inhibitory and stimulatory effect of bLf on the biofilm formation of *M. haemolytica* A2.** (**a**) Bacteria were pre-incubated overnight with 3.5 and 6 μM bLf, then transferred to a microplate for biofilm formation; after 48 h, bacteria that had previous contact with bLf were no longer able to form a biofilm as bacteria without treatment do, since there was a reduction of 38 and 51% with incubation of 3.5 and 6 μM bLf, respectively. (**b**) Bacteria culture was transferred to a microplate for biofilm formation, and 3.5 and 6 μM bLf were added; after 48 h, an increase in biofilm was observed compared to the biofilm of untreated bacteria. These results were dependent on bLf concentration, as a significant difference was obtained between the different bLf concentrations used; bovine Lf alone was added to the microplate without biofilm to discard adherence of bLf to the microplate. Statistically significant differences between ratios are indicated (* *p* < 0.05, ** *p* < 0.01, *** *p* < 0.001). Representative results of three independent experiments.

To determine the ability of *M. haemolytica* A2 to form biofilm when grown with bLf, bacteria were preincubated overnight with sub-inhibitory concentrations of bLf (3.5 and 6 μM); then, bacteria were transferred to a 96-well plate for biofilm formation. In another assay, bacteria were grown overnight without bLf and then transferred to a 96-well plate where bLf was added. Bacteria pre-incubated with 3.5 and 6 μM bLf showed less biofilm formation (38 and 51%, respectively) compared to bacteria that had no previous contact with bLf (Figure 2a). However, when bLf was added during biofilm formation, an increase in biofilm was observed compared to a biofilm without bLf (Figure 2b). These results suggest that the effect of bLf on the biofilm is dependent on the stage at which it is added.

### 2.3. Bovine Lactoferrin Increases the Mature Biofilm of M. haemolytica A2

**Figure 3:**
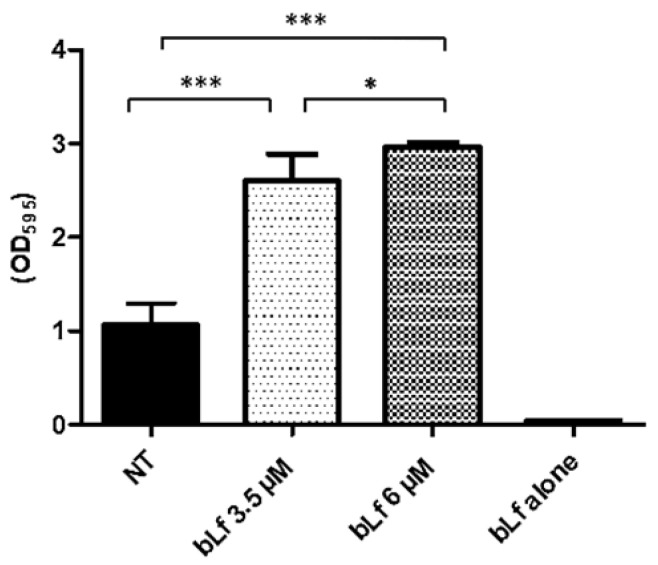
**Stimulatory effect of bLf on a mature biofilm of *M. haemolytica* A2**. Bovine Lf (3.5 and 6 μM) was added to a 48 h biofilm of *M. haemolytica* A2. After 24 h, an increase in biofilm with bLf was observed, compared to a biofilm of bacteria with no treatment. These results were dependent on bLf concentration, as a significant difference was obtained between both bLf concentrations used, respectively; Lf alone was added to the microplate without biofilm to discard adherence of bLf to the microplate. Statistically significant differences between ratios are indicated (* *p* < 0.05, *** *p* < 0.001). Representative results of three independent experiments.

Since other reports have shown the ability of bLf to disaggregate mature biofilms, we tested if there was a similar effect on the biofilm of this bacterium. To analyze the effect of bLf on a mature biofilm of *M. haemolytica* A2, 3.5 and 6 μM bLf were added to 48 h biofilms, which were left for another 24 h, and then quantification was achieved using the crystal violet assay. Surprisingly, biofilms where bLf was added were greater than biofilms with no bLf (Figure 3). Taken together, these results show that it is possible that bLf may cause a stress response and consequently increase the biofilm; on the other hand, bLf could also be adhered to the biofilm surface and cause an increase in its density.

### 2.4. Scanning Electron Microscopy

**Figure 4:**
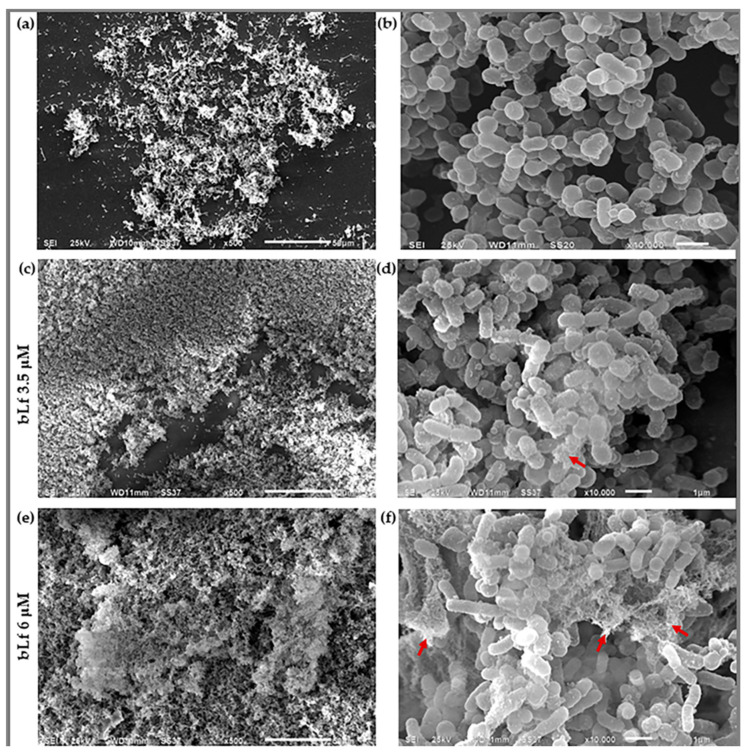
**Biofilm observation under a Scanning Electron Microscope.** Biofilm of *M. haemolytica* A2 with no treatment (NT) at a magnification of 500× (**a**) and 10,000× (**b**). The blank space shows the surface to which it is attached; biofilm with 3.5 μM bLf at a magnification of 500× (**c**) and 10,000× (**d**). The biofilm was observed to a greater extent and structures on the bacterial surface were noticed, compared to the biofilm with no treatment (red arrow). Biofilm with 6 μM bLf at a magnification of 500× (**e**) and 10,000× (**f**). A remarkable increase in biofilm was observed, indicated by the lack of blank space, as well as an increase in the structures on the bacterial surface and a mesh-like coating (red arrows). Representative images of three independent samples.

Since the increase in biofilm was observed when bLf was added at the beginning of biofilm formation, we continued analyzing the effect of this glycoprotein on biofilms at this stage. To this end, we conducted the following experiments. We added bLf to the microtiter plates and allowed biofilm formation for 48 h. After that, biofilms were evaluated. First, the increase in biofilms when bLf was added was corroborated by SEM. The images show an increase in bacteria aggregation adhered to the surface, indicated by the presence of components in the biofilm where bLf was added (Figure 4c–f) compared to the biofilm with no treatment (Figure 4a,b). Aggregation may be due to those components that form a part of the biofilm matrix, or it may also be due to the lactoferrin attached to the biofilm. These results confirmed an increase in biofilm and show that the morphology of the biofilm is different when a stimulus such as bLf is present, compared to a biofilm without bLf.

### 2.5. Proteins and Carbohydrates Are Increased in Biofilms When bLf Is Added

**Figure 5:**
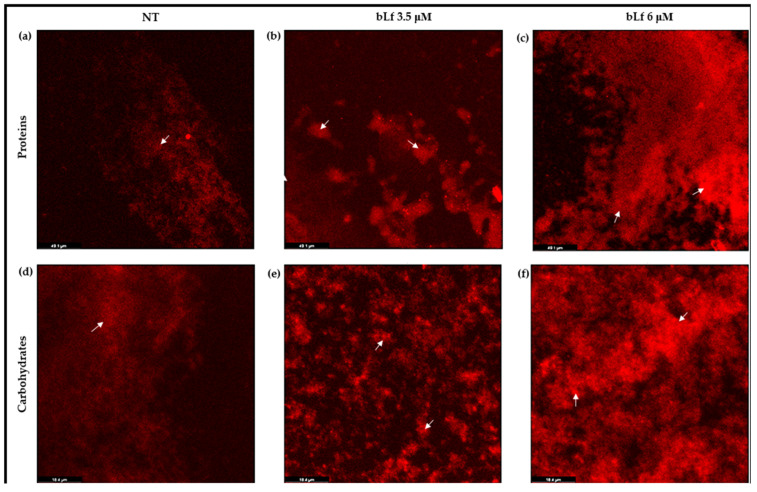
**Proteins and carbohydrates of the biofilm matrix of *M. haemolytica* A2, observed under a laser confocal microscope.** Images show proteins of the biofilm without bLf (NT) with the Sypro Ruby stain (**a**–**c**), and carbohydrates of the biofilm with the Red Texas stain (**d**–**f**); proteins and carbohydrates from biofilm incubated with 3.5 μM bLf (**b**,**e**) and 6 μM bLf (**c**,**f**) are shown. The intensity of the fluorescence (white arrows) in the biofilms with bLf shows a major amount of these components compared to the biofilm with no treatment. Image magnification: 40×. Representative images of three independent samples.

Proteins and carbohydrates are the main components of a biofilm of *M. haemolytica*. Therefore, if the biofilm is increasing in the presence of bLf, these components could be secreted in greater amounts compared to a biofilm without bLf. To visualize them, biofilms were stained and visualized under CLM, using Film Tracer^TM^ Sypro Ruby stain and Red Texas conjugated with Concanavalin A for proteins and carbohydrates, respectively. The images show the intensity of fluorescence for both components, proteins and carbohydrates, with a greater dispersion throughout the biofilm incubated with bLf (Figure 5b,c,e,f), compared to the biofilm where bLf was not added (Figure 5a,d). These results suggest that, in the presence of bLf, *M. haemolytica* does secrete components that will form the biofilm matrix to a greater extent than a biofilm where bLf is not added.

### 2.6. Protein Pattern of the Biofilm of M. haemolytica A2

**Figure 6:**
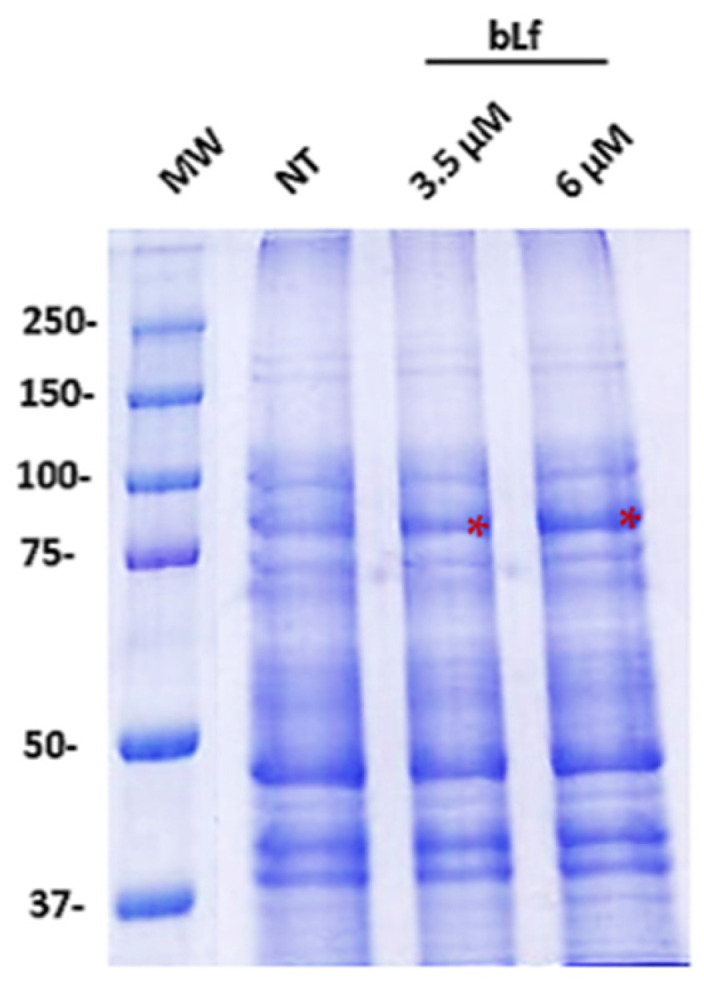
**Results of 10% SDS-PAGE of *M. haemolytica* A2 biofilm proteins incubated or unincubated with bLf.** The protein pattern was analyzed by SDS-PAGE, using samples of 48 h biofilms with or without 3.5 and 6 μM bLf. With this assay, we did not observe differences in the biofilm pattern when bLf was added, in comparison to the biofilm without bLf. In addition, a remarkable band corresponding to the molecular weight of bLf (~80 kDa) was observed where it was added (red asterisks). Representative results of three independent samples.

Since proteins are the major component of the biofilm matrix in *M. haemolytica*, we analyzed the proteins secreted in a 48 h biofilm with or without bLf through SDS-PAGE. We observed no difference in the protein pattern compared to the biofilm with no treatment (Figure 6). However, we observed a more remarkable band of approximately 80 kDa in the biofilm where bLf was added. With these results, we suggest the possible presence of bLf in the *M. haemolytica* biofilm.

### 2.7. Bovine Lactoferrin Is Present on the Biofilm of M. haemolytica A2

**Figure 7:**
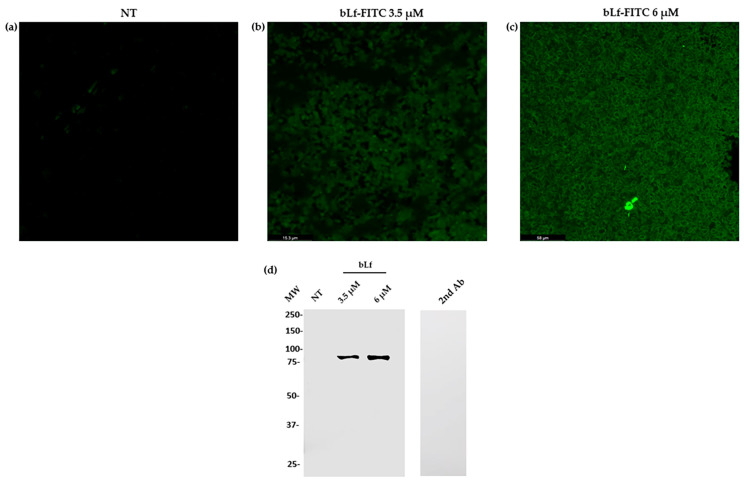
**Presence of bLf in the biofilm of *M. haemolytica* A2 shown through laser confocal microscopy and Western blot**. Images show the distribution of bLf labeled with FITC (green fluorescence) at 3.5 μM (**b**) and 6 μM (**c**) throughout the biofilm of *M. haemolytica* A2, and a negative control where FITC-bLf was added to the plate with no biofilm (**a**). Western blot using anti-bLf in a biofilm without bLf (NT), with 3.5 or 6 μM bLf and a secondary anti-rabbit-HRP antibody alone as a negative control (**d**). Representative results of three independent samples.

To corroborate the presence of bLf in the biofilm of *M. haemolytica* A2, CLM was first performed to visualize the distribution of bLf on the biofilm. To this end, bLf was FITC-labeled and added to the biofilms for 48 h. After this, biofilms were washed and observed under the microscope. The images show the distribution of 3.5 and 6 μM bLf on the biofilms (Figure 7b,c) and a negative control where bLf-FITC was added to the plate without biofilm (Figure 7a). In another assay, biofilms were grown for 48 h in a 96-well plate with or without bLf, and then the wells were washed and the biofilms recovered. With these samples, a Western blot assay was carried out using a rabbit anti-bLf serum. The results show a signal in the biofilm samples where bLf was added (Figure 7d). Taken together, these results show that bLf is present in the biofilm of *M. haemolytica* A2. In addition, there was an increase in the components of the biofilm matrix when bLf was added, and there was also an increase due to the presence of bLf in the biofilms.

### 2.8. Viability of M. haemolytica A2 Cells in Biofilms with bLf

**Figure 8:**
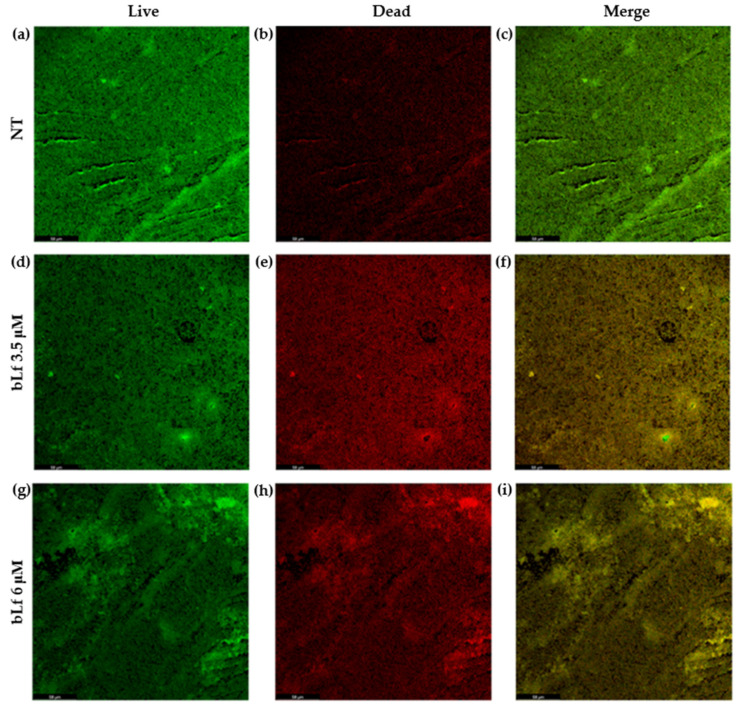
**Bovine Lf affects the viability of bacteria in biofilms with bLf, visualized by Confocal Laser Microscopy.** CLM images of bacteria in biofilm for 48 h without bLf (NT) (**a**–**c**) and with 3.5 (**d**–**f**) or 6 μM (**g**–**i**) bLf, showing live bacteria with the Sypro stain (**a**,**d**,**g**) and dead bacteria with the propidium iodide exclusion stain (**b**,**e**,**h**). The merge shows a greater distribution of dead bacteria (visualized by the distribution of yellow fluorescence) in a biofilm with bLf (**f**,**i**) than in a biofilm without treatment, where a greater distribution of live bacteria was observed (visualized by the distribution of green fluorescence) (**c**). Representative images of three independent samples.

The binding of bLf to the biofilm of *M. haemolytica* A2 could somehow affect the bacterial cells of the biofilm. To analyze if bLf affected the viability of the bacterial cells, biofilms with and without bLf were stained with live/dead staining. SYPRO9 (Figure 8a,d,g) and propidium iodide (Figure 8b,e,h) were added to 48 h biofilms to visualize live and dead bacteria, respectively, under a confocal laser microscope. The results show the distribution of live and dead bacteria throughout the biofilms by the intensity of green and red fluorescence, respectively. In the biofilm with bLf, dead bacteria are observed to a greater extent (Figure 8f,i) compared to the biofilm without bLf (Figure 8c), where most of the live bacteria are found. With these results, we showed that bLf does affect the viability of bacteria when it adheres to the biofilm.

### 2.9. Bovine Lactoferrin Affects the Formation of a New Biofilm Cycle in M. haemolytica A2

**Figure 9:**
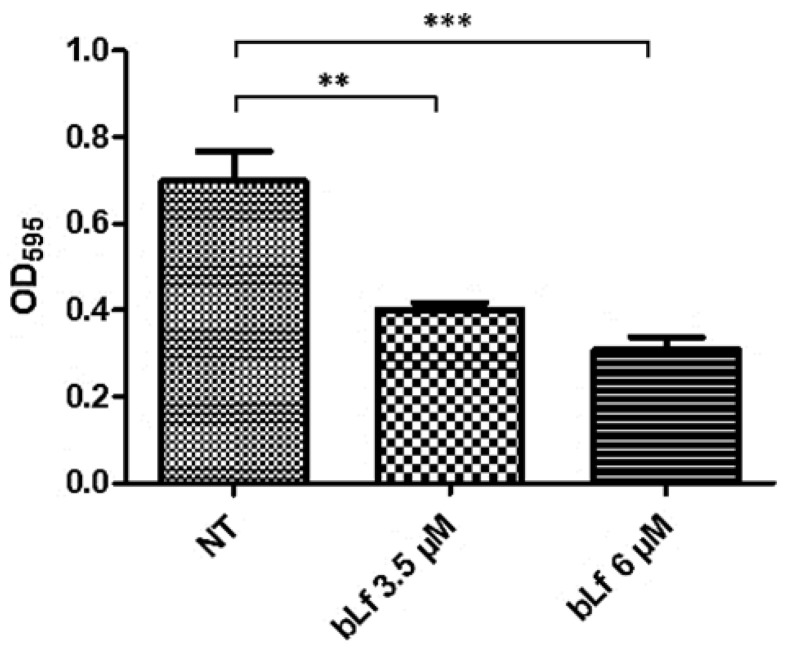
**Formation of a new biofilm cycle of *M. haemolytica* A2.** Bacteria of a 48 h biofilm with or without bLf were transferred to a new microplate to form a new biofilm cycle for another 48 h without bLf. A decrease in biofilm formation was observed in bacteria from a biofilm with bLf compared to bacteria from a biofilm with no treatment. Statistically significant differences between ratios are indicated (** *p* < 0.01, *** *p* < 0.001). Representative results of three independent experiments.

Once we determined that bLf could provoke the death of most of the bacteria in the biofilm, we tested if the rest of the bacteria that survive in the biofilm could form a new biofilm cycle, as the bacteria from a biofilm without bLf do. To this end, the biofilms were grown as before in a 96-well plate for 48 h with or without bLf, then harvested and transferred to a new medium without bLf, where they were left for 48 h. Later, biofilms were quantified by the crystal violet assay. The results show that bacteria from a biofilm with bLf formed significantly less biofilm than bacteria from a biofilm without bLf (Figure 9). This result suggests damage to the bacteria in the biofilm by the adherence of bLf, affecting this important mechanism of pathogenicity.

## 3. Discussion

*M. haemolytica* is a normal inhabiting bacterium of the upper respiratory tract of ruminants; however, under certain conditions, such as changes in temperature, stress, and infections with other pathogens, it is capable of colonizing and causing pneumonia [1,29]. *M. haemolytica* forms biofilms in vitro [12] and in vivo [30]. Biofilm formation helps bacteria survive harsh environments and protects them from the immune responses of the host, bacteriophages, and some antibiotics [10,31]. Lactoferrin, a glycoprotein of the innate immune system of mammals, has shown an inhibitory effect on biofilm formation in different bacteria such as *Pseudomonas aeruginosa* [22], *Streptococcus pneumoniae* [32], *Porphyromonas gingivalis* [19], and enteroaggregative *Escherichia coli* [21]. However, under some conditions, bLf increases biofilms, such as in *Streptococcus mutans* [24] and some strains of *Actinobacillus pleuropneumoniae* [25]. Here, we show the effect of sub-inhibitory concentrations of bLf on the biofilm formation of *M. haemolytica* A2, an ovine pathogen. Due to the interaction times of bLf with *M. haemolytica* in the biofilm assays being longer (48 h) than those reported before by our research group [27,28], we first tested the viability of the bacterium with different concentrations of this glycoprotein at times of 0, 16, 24, 36, and 48 h. In this analysis, we observed no difference in bacterial growth at concentrations of 3.5 and 6 μM in all experiments regarding the control. Then, for the following assays, the sub-inhibitory concentrations of bLf at 3.5 and 6 μM were used.

Then, we evaluated the effect of bLf on the biofilm with different conditions; in the first condition, bacteria were pre-incubated overnight with bLf (3.5 and 6 μM) or left unincubated, then transferred to the microtiter plate to form a biofilm for 48 h. Results show that bacteria that had previous contact with bLf formed less biofilm compared to the control bacteria; it has been demonstrated that bLf can inhibit the biofilm formation on Gram-negative bacteria through its iron chelating ability [23] or by its ability to act as a DNAase, disrupting the DNA present in the biofilm matrix in *Streptococcus pneumoniae* [32]. In addition, it is known that bLf binds to the components of bacterial membranes, causing damage and inhibiting the bacteria’s attachment to the surface [33]. In *M. haemolytica* A2, our group has reported that sub-inhibitory concentrations of bLf can damage the bacterial membrane [27]. Therefore, we suggest that when bLf is pre-incubated during bacterial growth, bLf could bind bacteria, affecting the ability of *M. haemolytica* to start the process of biofilm formation. 

Since bLf has shown an effect during the process of biofilm formation and on mature biofilms in other bacteria, [22,32], we analyzed the effect of bLf during the process of biofilm formation and on a mature biofilm of *M. haemolytica* A2. Surprisingly, in these conditions, bLf does not decrease either during the process of biofilm formation or on the mature biofilm; however, it does increase the biofilms. This increase was also observed under SEM, where we also noticed a different morphology and differences in the structure of the biofilms where bLf was added, which was dependent on the concentration of this glycoprotein. This result agrees with other reports where the stimulatory effect of bLf on biofilm formation in *A. pleuroneumoniae* [25] and *S. mutans* [24] are shown. Additionally, it has been reported that bLf binds to *Clostridium* cells and promotes agglutination by forming a complex [34]. 

The main step of biofilm formation is the production of the extracellular matrix by bacteria, which is mainly composed of proteins, eDNA, polysaccharides, lipids, and water molecules [35]. To know if bLf increases biofilm formation by increasing the secretion of components of the biofilm matrix, we determined the secretion of proteins and carbohydrates, two main components of the biofilm of *M. haemolytica* A2. To this end, biofilms were obtained and stained with Sypro and Red Texas to observe proteins and carbohydrates, respectively; an increase in these components was observed in the presence of bLf compared to the control. In addition, to evaluate if the secretion of different proteins for biofilm formation occurred in the presence of bLf, we analyzed the protein pattern of biofilms with or without bLf by SDS-PAGE. The results showed no difference in the protein pattern in all conditions, but a remarkable band of approximately 80 kDa was observed only in biofilms where bLf was added, suggesting the presence of bLf attached to the biofilm. With our results, we conclude that in the presence of bLf, *M. haemolytica* A2 secretes proteins and carbohydrates in greater amounts than bacteria with no bLf, and the proteins secreted were not different between both conditions. Since biofilms are a mechanism of protection, it is proposed that stress factors trigger the production of biofilms, and bLf could cause a similar response to protect itself from this antibacterial glycoprotein. Additionally, it is known that bLf can adhere to components of the bacterial membrane, such as outer membrane proteins (OmpA and porins) [36,37]. Since the biofilm of *M. haemolytica* is mainly composed of proteins, our results also suggest that in addition to provoking the secretion of more components of the biofilm matrix, bLf could be attached to the bacterial membrane or to the biofilm, leading to an increase in this structure. Therefore, through CLM and Western blot assays, the presence of bLf on the biofilm was confirmed. It is suggested that the biofilm matrix can limit the diffusion of antimicrobial components through direct contact. However, in *P. aeruginosa*, it has been reported that Lf is not only present but also penetrates the biofilm. Due to this, bLf could also interact with bacteria embedded in biofilms [38]. Since bLf has shown binding to outer membrane proteins from *M. haemolytica* through its N-terminal, which is responsible for the antimicrobial effect of bLf, we do not discard this possibility. Additionally, the fact that the biofilm matrix of *M. haemolytica* is mainly composed of proteins, it is suggested that bLf may also be interacting with the biofilm matrix. Regarding this, we proposed that bLf could increase the biofilm of *M. haemolytica* A2 by increasing bacterial aggregation and secretion of components of the biofilm matrix, as well as by the attachment of glycoproteins to the biofilm.

The attachment of bLf to the biofilm of *M. haemolytica* A2 could possibly cause damage to the community of bacteria. To analyze this possibility, the viability of bacteria in a biofilm with bLf was tested under CLM with live/dead staining; the results showed that most of the bacteria in a biofilm with bLf were dead, compared to bacteria in a biofilm without bLf, where most of the bacteria were observed to be living. A similar result was observed in another report, where it was shown that Lf chimera (a fusion peptide obtained from lactoferricin and lactoferrampin) affects bacterial viability in oral biofilms, since most of the bacteria in the biofilm were observed to be dead, compared to the control [39]. With this result, we suggest that bLf may be affecting bacteria in the biofilm by blocking the entrance of nutrients and oxygen by binding to the biofilm matrix, or it may be disrupting the bacterial membrane by direct contact, affecting its permeability and bacterial mechanisms of pathogenicity, leading to bacterial death. 

Bacterial biofilm formation is a multi-step process that does not end at the dispersion step. Bacteria that detach from the biofilm adhere to a new surface to continue the biofilm process [40,41]. Since we also observed some live bacteria, we continued analyzing if the surviving bacteria were able to form a new biofilm cycle. To this end, bacteria from a biofilm with or without bLf were transferred to a new plate to form a new biofilm for 48 h, and the biofilm growth was measured by the crystal violet assay. It was observed that bacteria from a biofilm with bLf formed fewer new biofilms than bacteria from a biofilm without bLf, suggesting that detached bacteria will not be able to colonize another surface and could be killed easily in their planktonic state. Taken together, these results show that bLf forms part of the biofilm and affects the viability of *M. haemolytica* A2 cells in the biofilm, as well as the formation of a new biofilm cycle. 

## 4. Materials and Methods

### 4.1. Bacterial Strain and Culture

A field strain of *M. haemolytica* A2 isolated from a lamb with pneumonia and identified by PCR in our previous work [42] was used in this study. The strain was identified by API 20E (bioMérieux, Mexico City, Mexico). *M. haemolytica* A2 was cultured on blood agar at 37 °C for 24 h before being used in each experiment. 

### 4.2. Viability of M. haemolytica A2 in the Presence of Bovine Lactoferrin

The effect of bLf on the growth of *M. haemolytica* A2 was evaluated at different incubation times (16, 24, 36, and 48 h) and concentrations of apo-bLf (Nutriscience Innovations, Milford, CT, USA, 97% purity, containing 5 mg of iron per 100 g protein), ranging from 3.5 to 9 μM. To this end, the microdilution method was applied [25]. Briefly, bacteria (10^6^ CFU) were incubated in BHI medium (Dibico, Mexico City, Mexico) with or without bLf and incubated at 37 °C with agitation (150 rpm). After each incubation period, bacteria were recovered, diluted in saline solution, and subsequently plated on BHI agar. Plates were then incubated for 24 h at 37 °C, and the colony-forming units (CFUs) were counted.

### 4.3. Effect of Bovine Lactoferrin on Biofilm Formation of M. haemolytica A2

Bacterial cultures were grown in BHI medium (Dibico) for 24 h at 37 °C with agitation (150 rpm) with or without bLf at 3.5 or 6 μM. Then, bacteria (10^6^ CFU) were transferred to 200 μL of BHI medium in a sterile 96-well microtiter plate (Corning, Kennebunk, ME, USA) and incubated for 48 h at 37 °C with slow agitation (90 rpm). In a second assay, we evaluated the effect of bLf on the biofilm formation of *M. haemolytica* A2; to this end, bacteria were grown as described before without bLf, then transferred to the sterile 96-well microtiter plate (Corning, Kennebunk, ME, USA). bLf at 3.5 or 6 μM was added and the plate was incubated for 48 h at 37 °C with agitation (90 rpm). Finally, to evaluate the effect of bLf on the mature biofilm, bacteria were grown, and the biofilm assay was performed as described before but without bLf. Then, after 48 h, bLf at 3.5 and 6 μM was added to the plate and incubated for 24 h at 37 °C with agitation (90 rpm). After each incubation period, the medium was decanted, and the wells were washed to eliminate non-adherent bacteria. The excess water was removed, and crystal violet (0.1%) (Sigma-Aldrich, Saint Louis, MO, USA) was added. Then, the plate was incubated for 15 min at room temperature (23 °C). The plate was washed three times, the excess water was removed, and 200 μL of 70% ethanol was added to each well. Absorbance was measured at 595 nm (ELx808 microplate reader) [43]. 

### 4.4. Morphological Observation

To visualize the biofilm’s morphology, observation under scanning electron microscopy (SEM) was carried out. To this end, a 48 h biofilm with or without bLf was grown on a 0.5 × 0.5 cm glass slide (Corning, Kennebunk, ME, USA). Afterwards, it was fixed in 2.5% glutaraldehyde (Sigma-Aldrich, Saint Louis, MO, USA) for 1 h at room temperature (23 °C) with gentle agitation. The sample was then washed with 0.1 M cacodylate buffer at pH 7.2 (Sigma-Aldrich, Saint Louis, MO, USA) with gentle agitation. Next, 1% osmium tetroxide (Sigma-Aldrich, Saint Louis, MO, USA) was added for 1 h at room temperature (23 °C) with gentle agitation and washed as described before. After that, the samples were dehydrated with ethanol gradients (50%, 60%, 70%, 80%, 90%, and 100%) for 10 min each, and prepared for observation using a scanning electron microscope at 25 kV (Thermo Scientific, Waltham, MA, USA) [44].

### 4.5. Biofilm Macromolecular Analysis

The macromolecules in a 48 h biofilm with or without bLf were analyzed. Biofilms were grown in a sterile polystyrene chamber (Corning, Kennebunk, ME, USA). To visualize proteins, samples were stained with 200 μL of FilmTracerTM SYPRO^®^ Ruby (Invitrogen, Waltham, MA, USA) per well and incubated at room temperature (23 °C) for 30 min. For carbohydrates, samples were stained with Texas Red conjugated with concanavalin A (2.5 μg/mL) (Thermo Fisher, Waltham, MA, USA) at 37 °C for 30 min. The stains were added without fixation, according to the manufacturer’s instructions. All samples were observed under a confocal laser microscope (DMi8, Leica) with a 40× objective with fluorescence excitation/emission of 450/610 nm for the SYPRO Ruby biofilm matrix and 595/615 nm for Texas Red. The biofilm architectures were analyzed using LASX Software Version 3.7.5.24914.

### 4.6. SDS-PAGE

The protein pattern of a 48 h biofilm with or without bLf was analyzed using 10% polyacrylamide gels. Biofilms were grown in a 96-well plate (Corning, Kennebunk, ME, USA) as described before. Afterward, the medium was eliminated, and the wells were washed three times with PBS. Biofilms were collected and centrifuged at 1800× *g* for 3 min, and the pellet was washed and resuspended in PBS1X, mixed on a vortex, and centrifuged at 12,000× *g* for 3 min. Protein quantification of the liquid phase was achieved by the Bradford method [45]. The protein concentration of the samples was adjusted to 10 μg for electrophoresis, which was performed under the following conditions: S1: 10 min at 80 volts; S2: 2: 30 h at 100 volts, at 4 °C. After electrophoresis, the gels were stained with 0.5% (*w*/*v*) Coomassie brilliant blue R-250 (Bio-Rad, Feldkirchen, Germany).

### 4.7. Western Blot

The presence of bLf in the biofilms was determined through Western blot. To this end, biofilms were obtained, and 10% SDS-PAGE was performed as described before. Then, proteins were transferred to a nitrocellulose membrane (Sigma-Aldrich) for 1 h at 400 mA at 4 °C. The membrane was blocked with skim milk for 1 h at room temperature (23 °C) and then incubated overnight with a rabbit anti-bLf (1:5000) at 4 °C. After incubation, the membrane was washed three times with PBS/Tween (0.05%) (Sigma-Aldrich, St. Louis, MO, USA) and incubated with a secondary anti-rabbit-HRP antibody (1:3000) (Sigma) for 1 h at 4 °C. The membrane was then washed as mentioned above and developed with a luminol kit reagent (Santa Cruz Biotechnology, Dallas, Germany). Images were obtained using the Odyssey FC imaging system (LI-COR) [42]

### 4.8. FITC-Labelled bLf and Laser Confocal Microscopy

Bovine Lf was prepared in sodium bicarbonate and resuspended in a carbonate buffer with agitation. Fluorescein isothiocyanate (FITC) (Sigma-Aldrich) was added to the solution and left in agitation for 2 h at room temperature (23 °C). Then, it was centrifuged at 5000× *g* for 15 min and dialyzed in PBS for 48 h. Biofilms were grown in a sterile polystyrene chamber where FITC-bLf was either added or not. After 48 h, the medium was eliminated, and the biofilms were washed three times with PBS and fixed with 4% paraformaldehyde (Sigma-Aldrich) for 30 min. Samples were then washed and prepared for CLM observation. Images were obtained using a 40× objective and analyzed with LASX Software Version 3.7.5.24914 [46].

### 4.9. Viability of Bacteria in a Biofilm with bLf

Bacteria were pre-incubated without bLf and transferred to the sterile polystyrene chamber where bLf was added. After 48 h, biofilms were obtained and washed with PBS 1X to eliminate planktonic bacteria. Biofilm cell viability was analyzed using live/dead staining; SYTO9 (Thermo Fisher, Waltham, MA, USA) was used to observe live bacteria, and propidium iodide (Thermo Fisher, Waltham, MA, USA) to observe dead bacteria. Samples were incubated at 37 °C for 20 min while protected from light, gently washed three times, and prepared for LCM observation. Images were obtained using a 63× objective and analyzed with LASX Software Version 3.7.5.24914 [39].

### 4.10. Formation of a New Biofilm Cycle

Bacterial cells were obtained from a biofilm in a 96-well plate (Corning, Kennebunk, ME, USA) after 48 h of growth with or without bLf, as described above, and transferred to a new 96-well plate without bLf to initiate a new biofilm cycle for 48 h at 37 °C. Afterwards, biofilms were quantified using the crystal violet assay, as mentioned before.

### 4.11. Statistical Analysis

Data were plotted as the mean of triplicate SD. The data obtained were analyzed by the software 9.2.0 GraphPad prism (GraphPad, CA, USA), by a one-way ANOVA study, and a Tukey’s test was performed; comparisons of * *p* < 0.05 were considered statistically significant. 

## 5. Conclusions

Our data show that the growth of *M. haemolytica* A2 with bLf affects the ability of bacteria to form biofilms. Furthermore, bLf affects the viability of bacterial cells embedded in the biofilm and suggests that the binding of bLf could be the mechanism by which it causes damage. Based on these findings, we propose the use of bLf to prevent or combat biofilm-associated infections of *M. haemolytica* A2 in ovine mannheimiosis. However, in vivo studies are necessary to confirm these effects against this mechanism of pathogenicity.

## Data Availability

Data are contained within the article.

## References

[1] Hussain R., Mahmood F., Ali H.M., Siddique A.B. (2017). Bacterial, PCR and Clinico-Pathological Diagnosis of Naturally Occurring Pneumonic Pasturellosis (Mannheimiosis) during Subtropical Climate in Sheep. Microb. Pathog..

[2] Abdulkadir M., Nigussie T., Kebede I.A. (2024). Isolation and Identification of *Pasteurella multocida* and *Mannheimia haemolytica* from Pneumonic Small Ruminants and Their Antibiotic Susceptibility in Haramaya District, Eastern Ethiopia. Sci. World J..

[3] Singh K., Ritchey J.W., Confer A.W. (2011). *Mannheimia haemolytica*: Bacterial-Host Interactions in Bovine Pneumonia. Vet. Pathol..

[4] Highlander S.K. (2001). Molecular Genetic Analysis of Virulence in *Mannheimia* (*pasteurella*) *haemolytica*. Front. Biosci..

[5] het Lam J., Derkman T.H.J., van Garderen E., Dijkman R., van Engelen E. (2023). Distinct *Mannheimia haemolytica* Serotypes Isolated from Fatal Infections in Veal Calves and Dairy Cows. Vet. J..

[6] Straus D.C., Jolley W.L., Purdy C.W. (1993). Characterization of Neuraminidases Produced by Various Serotypes of *Pasteurella haemolytica*. Infect. Immun..

[7] Zecchinon L., Fett T., Desmecht D. (2005). How *Mannheimia haemolytica* Defeats Host Defence through a Kiss of Death Mechanism. Vet. Res..

[8] Ramírez Rico G., Martínez-Castillo M., González-Ruíz C., Luna-Castro S., de la Garza M. (2017). *Mannheimia haemolytica* A2 Secretes Different Proteases into the Culture Medium and in Outer Membrane Vesicles. Microb. Pathog..

[9] Kragh K.N., Tolker-Nielsen T., Lichtenberg M. (2023). The Non-Attached Biofilm Aggregate. Commun. Biol..

[10] Yin W., Wang Y., Liu L., He J. (2019). Biofilms: The Microbial “Protective Clothing” in Extreme Environments. Int. J. Mol. Sci..

[11] Venkatesan N., Perumal G., Doble M. (2015). Bacterial Resistance in Biofilm-Associated Bacteria. Future Microbiol..

[12] Boukahil I., Czuprynski C.J. (2015). Characterization of *Mannheimia haemolytica* Biofilm Formation in Vitro. Vet. Microbiol..

[13] Roy R., Tiwari M., Donelli G., Tiwari V. (2018). Strategies for Combating Bacterial Biofilms: A Focus on Anti-Biofilm Agents and Their Mechanisms of Action. Virulence.

[14] Hemmati F., Rezaee M.A., Ebrahimzadeh S., Yousefi L., Nouri R., Kafil H.S., Gholizadeh P. (2021). Novel Strategies to Combat Bacterial Biofilms. Mol. Biotechnol..

[15] Abdelhamid A.G., Yousef A.E. (2023). Combating Bacterial Biofilms: Current and Emerging Antibiofilm Strategies for Treating Persistent Infections. Antibiotics.

[16] Grooters K.E., Ku J.C., Richter D.M., Krinock M.J., Minor A., Li P., Kim A., Sawyer R., Li Y. (2024). Strategies for Combating Antibiotic Resistance in Bacterial Biofilms. Front. Cell. Infect. Microbiol..

[17] Hall C.W., Mah T.F. (2017). Molecular Mechanisms of Biofilm-Based Antibiotic Resistance and Tolerance in Pathogenic Bacteria. FEMS Microbiol. Rev..

[18] Avalos-Gómez C., Ramírez-Rico G., Ruiz-Mazón L., Sicairos N.L., Serrano-Luna J., de la Garza M. (2022). Lactoferrin: An Effective Weapon in the Battle Against Bacterial Infections. Curr. Pharm. Des..

[19] Dashper S.G., Pan Y., Veith P.D., Chen Y.Y., Toh E.C.Y., Liu S.W., Cross K.J., Reynolds E.C. (2012). Lactoferrin Inhibits *Porphyromonas gingivalis* Proteinases and Has Sustained Biofilm Inhibitory Activity. Antimicrob. Agents Chemother..

[20] Wakabayashi H., Yamauchi K., Kobayashi T., Yaeshima T., Iwatsuki K., Yoshie H. (2009). Inhibitory Effects of Lactoferrin on Growth and Biofilm Formation of *Porphyromonas gingivalis* and *Prevotella intermedia*. Antimicrob. Agents Chemother..

[21] Ochoa T.J., Brown E.L., Guion C.E., Chen J.Z., McMahon R.J., Cleary T.G. (2006). Effect of Lactoferrin on Enteroaggregative *E. coli* (EAEC). Biochem. Cell. Biol..

[22] Kamiya H., Ehara T., Matsumoto T. (2012). Inhibitory Effects of Lactoferrin on Biofilm Formation in Clinical Isolates of *Pseudomonas aeruginosa*. J. Infect. Chemother..

[23] Lu J., Francis J.D., Guevara M.A., Moore R.E., Chambers S.A., Doster R.S., Eastman A.J., Rogers L.M., Noble K.N., Manning S.D. (2021). Antibacterial and Anti-Biofilm Activity of the Human Breast Milk Glycoprotein Lactoferrin against Group B *Streptococcus*. ChemBioChem.

[24] Francesca B., Ajello M., Bosso P., Morea C., Andrea P., Giovanni A.-O., Piera V. (2004). Both Lactoferrin and Iron Influence Aggregation and Biofilm Formation in *Streptococcus mutans*. Biometals.

[25] Luna-Castro S., Aguilar-Romero F., Samaniego-Barrón L., Godínez-Vargas D., De La Garza M. (2014). Effect of Bovine Apo-Lactoferrin on the Growth and Virulence of *Actinobacillus pleuropneumoniae*. BioMetals.

[26] Ojima Y., Nunogami S., Taya M. (2016). Antibiofilm Effect of Warfarin on Biofilm Formation of *Escherichia coli* Promoted by Antimicrobial Treatment. J. Glob. Antimicrob. Resist..

[27] Avalos-Gómez C., Reyes-López M., Ramírez-Rico G., Díaz-Aparicio E., Zenteno E., González-Ruiz C., De La Garza M. (2020). Effect of Apo-Lactoferrin on Leukotoxin and Outer Membrane Vesicles of *Mannheimia haemolytica* A2. Vet. Res..

[28] Ramírez-Rico G., Martinez-Castillo M., Avalos-Gómez C., de la Garza M. (2021). Bovine Apo-Lactoferrin Affects the Secretion of Proteases in *Mannheimia haemolytica* A2. Access Microbiol..

[29] Scott P.R. (2011). Treatment and Control of Respiratory Disease in Sheep. Vet. Clin. North Am.—Food Anim. Pract..

[30] Boukahil I., Czuprynski C.J. (2016). *Mannheimia haemolytica* Biofilm Formation on Bovine Respiratory Epithelial Cells. Vet. Microbiol..

[31] Yan J., Bassler B.L. (2019). Surviving as a Community: Antibiotic Tolerance and Persistence in Bacterial Biofilms. Cell Host Microbe.

[32] Angulo-Zamudio U.A., Vidal J.E., Nazmi K., Bolscher J.G.M., Leon-Sicairos C., Antezana B.S., Canizalez-Roman A., León-Sicairos N. (2019). Lactoferrin Disaggregates Pneumococcal Biofilms and Inhibits Acquisition of Resistance Through Its DNase Activity. Front. Microbiol..

[33] Ammons M.C., Copié V. (2013). Mini-Review: Lactoferrin: A Bioinspired, Anti-Biofilm Therapeutic. Biofouling.

[34] Tomita S., Hagiwara K., Matsuyama J., Kiyosawa I. (1998). Binding of Lactoferrin to Bacterial Cells of the *Clostridium* Species and Their Agglutination. Biosci. Biotechnol. Biochem..

[35] Rather M.A., Gupta K., Mandal M. (2021). Microbial Biofilm: Formation, Architecture, Antibiotic Resistance, and Control Strategies. Braz. J. Microbiol..

[36] Erdei J., Forsgren A., Naidu A.S. (1994). Lactoferrin Binds to Porins OmpF and OmpC in *Escherichia coli*. Infect. Immun..

[37] Samaniego-Barrón L., Luna-Castro S., Piña-Vázquez C., Suárez-Güemes F., De La Garza M. (2016). Two Outer Membrane Proteins Are Bovine Lactoferrin-Binding Proteins in *Mannheimia haemolytica* A1. Vet. Res..

[38] Ammons M.C.B., Ward L.S., Fisher S.T., Wolcott R.D., James G.A. (2009). In Vitro Susceptibility of Established Biofilms Composed of a Clinical Wound Isolate of *Pseudomonas aeruginosa* Treated with Lactoferrin and Xylitol. Int. J. Antimicrob. Agents.

[39] Ruangcharoen S., Suwannarong W., Lachica M.R.C.T., Bolscher J.G.M., Nazmi K., Khunkitti W., Taweechaisupapong S. (2017). Killing Activity of LFchimera on Periodontopathic Bacteria and Multispecies Oral Biofilm Formation in Vitro. World J. Microbiol. Biotechnol..

[40] Guilhen C., Forestier C., Balestrino D. (2017). Biofilm Dispersal: Multiple Elaborate Strategies for Dissemination of Bacteria with Unique Properties. Mol. Microbiol..

[41] Zhao A., Sun J., Liu Y. (2023). Understanding Bacterial Biofilms: From Definition to Treatment Strategies. Front. Cell. Infect. Microbiol..

[42] Ramírez-Rico G., Martinez-Castillo M., Ruiz-Mazón L., Meneses-Romero E.P., Palacios J.A.F., Díaz-Aparicio E., Abascal E.N., de la Garza M. (2024). Identification, Biochemical Characterization, and In Vivo Detection of a Zn-Metalloprotease with Collagenase Activity from *Mannheimia haemolytica* A2. Int. J. Mol. Sci..

[43] O’Toole G.A. (2010). Microtiter Dish Biofilm Formation Assay. J. Vis. Exp..

[44] Shatila F., Yaşa İ., Yalçın H.T. (2021). Biofilm Formation by *Salmonella enterica* Strains. Curr. Microbiol..

[45] Olson B.J.S.C. (2016). Assays for Determination of Protein Concentration. Curr. Protoc. Pharmacol..

[46] Chaganti L.K., Venkatakrishnan N., Bose K. (2018). An Efficient Method for FITC Labelling of Proteins Using Tandem Affinity Purification. Biosci. Rep..

